# The Indonesian Young-Adult Attachment (IYAA): An audio-video dataset for behavioral young-adult attachment assessment

**DOI:** 10.1016/j.dib.2023.109599

**Published:** 2023-09-21

**Authors:** Tusty Nadia Maghfira, Adila Alfa Krisnadhi, T. Basaruddin, Sri Redatin Retno Pudjiati

**Affiliations:** a Computer Science Department, Universitas Indonesia, Depok 16424 Indonesia; b Psychology Department, Universitas Indonesia, Depok 16424 Indonesia

**Keywords:** Attachment system, Affective computing, Emotion, Facial expression, Multi-modal, Social signal processing, Speech

## Abstract

The attachment system is an innate human instinct to gain a sense of security as a form of self-defense from threats. Adults with secure attachment can maintain the balance of their relationships with themselves and significant others such as parents, romantic partners, and close friends. Generally, the adult attachment assessment data are collected primarily from subjective responses through questionnaires or interviews, which are closed to the research community. Attachment assessment from behavioral traits has also not been studied in depth because attachment-related behavioral data are still not openly available for research. This limits the scope of attachment assessment to new alternative innovations, such as the application of machine learning and deep learning-based approaches. This paper presents the Indonesian Young Adult Attachment (IYAA) dataset, a facial expression and speech audio dataset of Indonesian young adults in attachment projective-based assessment. The assessment contains two stages: exposure and response of 14 attachment-based stimuli. IYAA consists of audio-video data from age groups between 18-29 years old, with 20 male and 67 female subjects. It contains 1216 exposure videos, 1217 response videos, and 1217 speech response audios. Each data has a varying duration; the duration for exposure video ranges from 25 seconds to 1 minute 39 seconds, while for response video and speech response audio ranges from 40 seconds to 8 minutes and 25 seconds. The IYAA dataset is annotated into two kinds of labels: emotion and attachment. First, emotion labeling is annotated on each stimulus for all subject data (exposure videos, response videos, speech response audios). Each data is annotated into one or more labels among eight basic emotion categories (neutral, happy, sad, contempt, anger, disgust, surprised, fear) since each attachment-related event involves unconscious mental processes characterized by emotional changes. Second, each subject is annotated into one among three attachment style labels: secure, insecure-anxious, and insecure-avoidance. Given these two kinds of labeling, the IYAA dataset supports several research purposes, either using one kind of label separately or using them together for attachment classification research. It also supports innovative approaches to build automatic attachment classification through collaboration between the study of Behavioral, Developmental, and Social Psychology with Social Signal Processing.

Specifications Table

**Table:** 

Subject	Computer Science

Specific subject area	Computer Vision, Speech Emotion Recognition, Affective Computing, Social Signal Processing
Type of data	Exposure Video, Response Video: Video files Response Speech Audio: Audio files Questionnaire Response: Table
How the data were acquired	Facial expression and speech responses were collected online using a video conference application, Zoom. Prior to video and audio data collection, each subject was requested to fill in the Experiences in Close Relationship-Relationship Structures (ECR-RS) questionnaire [1] to obtain the subject's perspective on his/her attachment style. Afterwards, each participant had a 1-on-1 Zoom session with one of our team members. For data collection purposes, each subject was instructed to prepare a laptop equipped with a working webcam and audio, the Zoom software installed, mics/headphones/headsets, and a smooth internet connection. The data collection session could take place in any location as long as participants ensured they were in a quiet room with bright light exposure and not accompanied by anyone. All assessments were recorded using the built-in recording feature of the Zoom software. After the data collection was done, the obtained video-audio recordings were edited using Davinci Resolve and Adobe Premiere Pro software.
Data format	Video: MPEG-4 (mp4). Audio: Waveform Audio File Format (wav). Cleansed and filtered.
Description of data collection	The IYAA dataset is an Indonesian young-adult behavioral response collected online using a projective-test-based attachment assessment procedure. The subjects are Indonesian young adults aged between 18-29 years old from various demographics. Originally, we collected data from 95 subjects, but 8 subjects’ data were excluded because they did not meet one or more points of the data validity criteria, both in terms of context and technical aspects. The criteria that need to be met by subjects during data collection include relevant responses to the stimuli and instructions given, quiet place with sufficient
	lighting, not accompanied by anyone, and a smooth internet connection. The IYAA dataset is multi-modal data that consists of facial expression videos, speech response audios, and questionnaire responses. The subjective responses are obtained from the ECR-RS questionnaire, which assesses attachment patterns in 4 specific relationships (i.e., mother, father, romantic partner, and close friend). The behavioral responses are obtained during the assessment procedure, divided into exposure and response. Each subject was exposed to 14 attachment-based theme stimuli, in the form of pictures, during exposure, and his/her facial expression response was recorded. This was then followed by the response stage, where each subject was given a minute to convey their feelings or experiences related to the activity or relationship in the presented picture. The subject's facial expressions and speech responses were recorded at this stage. Out of 87 subjects, 85 received a complete set of stimuli, from each of whom we obtained 14 exposure videos, 14 response videos, and 14 speech response audios. Due to technical issues, we failed to obtain a complete set of videos and audios from the remaining two subjects. From the first, we obtained a complete set of response videos and speech response audios, but only 13 exposure videos. From the second, we only obtained 13 of each of exposure videos, response videos, and speech response audios. In total, we obtained 1216 exposure videos, 1217 response videos, and 1217 speech response audios. Annotation information can be found in the included spreadsheet. Every subject is labeled as one of the following attachment styles: secure, anxious-insecure, and avoidance-insecure. In addition, each of the exposure videos, response videos, and speech response audios, is labeled with one or more basic emotion categories from neutral, happy, sad, contempt, anger, disgust, surprise, and fear.
Data source location	The online-based audio-visual attachment behavior responses data were conducted in Indonesia. The subjects came from various Indonesian ethnicities, including Javanese, Sundanese, Betawi, Madurese, Batak, Malay, Minang, Banjar, and Bugis.
Data accessibility	Repository name: ZenodoData identification number: 10.5281/zenodo.8127495[2]Direct URL to data: https://zenodo.org/record/8127495Since our data require access controls for ethical reasons, readers need to request access. The following are the criteria that need to be met to obtain the data access: 1. Fill out the access request form provided by Zenodo, by clicking on the “Request Access” button, then enter your full name, email address, and justification for requesting the dataset. 2. Fill out and sign the Data Use Agreement (DUA) attached to this article. Send the signed DUA to IYAA Dataset Development Team (iyaadata.devteam@gmail.com). We will evaluate and decide whether to grant access based on the information you submitted. If your access request meets the criteria, you will get two emails, 1) automated email from Zenodo notifying you that your access request is approved with a private link attached along with an expiration date, and 2) email from IYAA Dataset Development Team containing approved DUA.

## 1 Value of the Data

• The IYAA dataset is the first Indonesian young adult attachment behavioral dataset that:
  ○ is assessed based on adult's close relationship with self and specific attachment figures,
  ○ classifies attachment style for each subject based on his/her behavioral responses to attachment-related stimuli through facial expression videos and speech audios,
  ○ classifies emotions that appear through his/her responses regarding attachment-related stimuli.
• The IYAA dataset can be utilized to train either unimodal or multimodal machine learning models for attachment style classification tasks, including speech emotion recognition (SER) and facial emotion recognition (FER).
• The entire attachment assessment procedure is ethically approved and developed based on Bowlby and Ainsworth's attachment theory.
• The IYAA dataset can be used for study of attachment assessment specifically in the Behavior Psychology, Developmental Psychology, Social Psychology, and Social Signal Processing.

## 2 Objective

We purposefully collected the IYAA dataset as material for developing a machine learning-based attachment style classification model that focuses on behavioral traits of Indonesian young-adults. The study of machine learning-based attachment style classification focusing on behavioral traits has not been explored in depth due to the lack of publicly available attachment-related behavioral response datasets. The IYAA dataset, which consists of behavioral data in the form of facial expressions and speech responses, can also serve as an alternative assessment material to the questionnaires and interviews commonly used in attachment studies in Psychology.

## 3 Data Description

IYAA is a multi-modal dataset of behavioral and subjective responses related to the attachment system. Introduced by John Bowlby, attachment system is a manifestation of the human instinct to gain a sense of security as a self-defense mechanism against distress or threats [3]. When attachment system is activated, someone tends to display proximity seeking behavior towards his/her attachment figures, who act as a secure base and safe haven that provide protection, support, and comfort [4]. According to Bowlby, the relationship established between the infant and the primary attachment figure (usually the mother) becomes the basis mental representation that determines the relationship with other attachment figures throughout life (i.e., father, family members, friends, and romantic partners) [5].

The subjects who participated in this data collection are Indonesian young adults between 18-29 years old, with 20 male and 67 female subjects. The process of designing and collecting the IYAA dataset was carried out from 2021-2022 during the COVID-19 pandemic. Each subject was exposed with 14 pictures illustrating attachment-related events. We recorded his/her facial expression responses when the stimuli images were presented. Afterwards, each subject was asked to describe what (s)he felt about the pictures or any past experiences (s)he recalled when the pictures were shown. We thus collected 14 exposure videos, 14 response videos, and 14 speech response audios for each subject. However, for two female subjects, we encountered technical difficulties during data collection, causing us to lose one exposure video for the first and 2 videos (exposure and response) and one audio from the second. So, in total, we obtained 1216 exposure videos, 1217 response videos, and 1217 speech response audios. We also collected data about subject's responses about their relationship with attachment figures in the Experiences in Close Relationships – Relationship Structures (ECR-RS) questionnaire [1].

There are two kinds of IYAA dataset labels: emotion and attachment style. The IYAA dataset can support attachment studies with varied specific topics as these two types of labels can be used independently. First, the emotion labeling is annotated in one or more labels among eight emotion labels based on Ekman's basic emotion theory: *happy, sad, contempt, anger, disgust, surprise, fear,* and *neutral* [6]*.* It is annotated for each stimulus of all data (exposure videos, response videos, and speech response audios), as each stimulus triggers a behavior change that reflexively shows a range of different emotions. A video/audio can be labeled as, for example, sad if there is a segment in the video/audio where the subject shows a sad expression, but we do not annotate where the segment is located or the specific time the expression appears. Table 1 presents a visual representation of the emotion labeling of each subject along with the corresponding quantities on all data.

**Table 1:** Illustration of the emotion labeling and its amount on each label.

Dataset: Exposure Video								
Subject	Neutral	Happy	Sad	Contempt	Anger	Disgust	Surprised	Fear
id1_exp1.mp4	1	1	0	0	0	0	0	0
Id1_exp2.mp4	1	0	1	0	0	0	0	0
⋮	⋮	⋮	⋮	⋮	⋮	⋮	⋮	⋮
id1_exp14.mp4	0	0	1	0	0	0	0	0
⋮	⋮	⋮	⋮	⋮	⋮	⋮	⋮	⋮
id87_exp1.mp4	0	0	1	0	0	0	0	0
id87_exp2.mp4	1	0	1	0	0	0	0	0
⋮	⋮	⋮	⋮	⋮	⋮	⋮	⋮	⋮
id87_exp14.mp4	1	0	0	0	0	0	0	0
Total	457	359	424	26	31	20	36	51

Dataset: Response Video								

Subject	Neutral	Happy	Sad	Contempt	Anger	Disgust	Surprised	Fear

id1_respv1.mp4	1	1	0	0	0	0	0	1
Id1_respv2.mp4	0	1	0	0	0	0	1	0
⋮	⋮	⋮	⋮	⋮	⋮	⋮	⋮	⋮
Id1_respv14.mp4	0	0	1	0	1	0	0	1
⋮	⋮	⋮	⋮	⋮	⋮	⋮	⋮	⋮
id87_respv1.mp4	1	1	0	0	0	0	0	1
id87_respv2.mp4	0	1	0	0	0	0	1	0
⋮	⋮	⋮	⋮	⋮	⋮	⋮	⋮	⋮
id87_respv14.mp4	0	0	1	0	1	0	0	1
Total	103	735	624	59	55	67	12	129

Dataset: Speech Response Audio								

Subject	Neutral	Happy	Sad	Contempt	Anger	Disgust	Surprised	Fear

id1_resps1.wav	1	1	0	0	0	0	0	1
Id1_resps2.wav	0	1	0	0	0	0	1	0
⋮	⋮	⋮	⋮	⋮	⋮	⋮	⋮	⋮
Id1_resps14.wav	0	0	1	0	1	0	0	1
⋮	⋮	⋮	⋮	⋮	⋮	⋮	⋮	⋮
id87_resps1.wav	1	1	0	0	0	0	0	1
id87_resps2.wav	0	1	0	0	0	0	1	0
⋮	⋮	⋮	⋮	⋮	⋮	⋮	⋮	⋮
id87_resps14.wav	0	0	1	0	1	0	0	1
Total	103	735	624	59	55	67	12	129

**Fig. 1:**
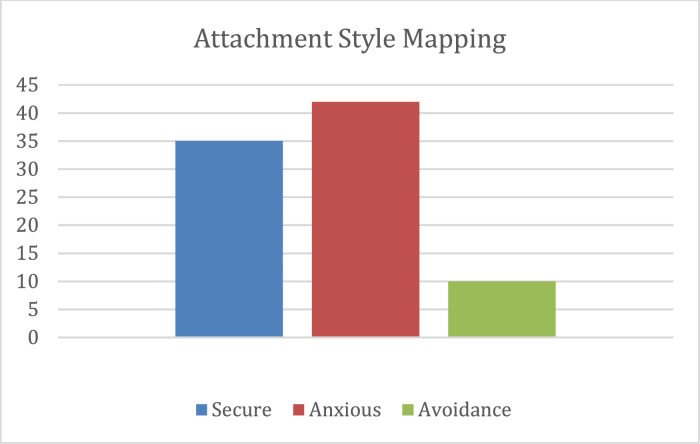
Attachment style mapping distribution.

The attachment style labeling categorizes each subject into one of three classes in Ainsworth's attachment mapping model: secure, insecure anxious, and insecure avoidance [7]. There are 35 subjects labeled as secure, 42 as insecure anxious, and 10 as insecure avoidance. Since each subject consists of multiple videos and audios data, this label can be viewed as the label of each subject's video/audio set. The distribution of subjects and their attachment labels is illustrated in Fig. 1.

Each data has been assigned a unique name as per our specification. The exposure videos are named as “id[subject]_exp[number-of-stimulus].mp4”, response videos are named as “id[subject]_respv[number-of-stimulus].mp4”, and speech response audios are named as “id[subject]_resps[number-of-stimulus].wav”. The word “id” defines a unique key for each subject, “exp” as abbreviation of exposure video, “respv” as abbreviation of response video, and “resps” as abbreviation of speech response audio.

Each data has a varying duration. The exposure video's duration ranges from 25 seconds to 1 minute 39 seconds. Data in stimulus raw fear and raw sadness has a longer duration than the other stimuli at 1 minute 30 seconds and 1 minute 39 seconds because they are displayed with supporting background music. For response video and speech response audio data have durations ranging from 40 seconds to 8 minutes and 25 seconds. In this response data, subjects who delivered responses of more than a minute were allowed to continue until they finished so that subjects could respond according to what they wanted to tell objectively and more comfortably without being rushed.

## 4 Experimental Design, Materials and Methods

The IYAA dataset collection procedure consists of stimuli selection, subject recruitment, self-report questionnaire completion, video and audio data collection, labeling and annotation. This section briefly describes each of these steps. All steps were conducted online by following health protocols during the COVID-19 pandemic. This dataset collection procedure has been ethically reviewed by the Ethics Review Team of the Department of Psychology Universitas Indonesia.

### 4.1 Stimuli selection

In this study, stimuli play an important role in triggering the subject to recollect their respective attachment relationships. The stimuli represent the internal working model of the self and significant attachment figures: mother, father, romantic partner, and close friend. The stimuli selection process is inspired by the study of the Biometric Attachment Test (BAT) [8], [9], [10], [11], [12], [13], [14], [15], [16], [17] by determining the stimuli theme rather than the fixed stimuli image. The theme concept acts as a placeholder for some images with the same functions. If we use the same fixed stimuli image set, people will no longer be triggered by it (priming effects). By using specific themes, we can give the same treatment for each subject and maintain dataset reusability for further study, either for cross-sectional or longitudinal study.

We proposed 14 stimuli themes in total, including: exploration, child solitude, raw sadness, raw fear, death or loss, vulnerable baby, attuned mother-child, attuned father-child, family connection, unresponsive mother, violent father, attuned couple, couple separation, attuned friendship. Among 14 stimuli themes, 13 of them adopted from [8] and one theme added is the close friend-related theme. The compilation of stimuli themes is inspired by the Separation Anxiety Test (SAT), Adult Attachment Projective (AAP), Strange Situation Procedure (SSP), ECR-RS, and attachment basic theory. In this study, we used the same stimuli themes set for all subjects. Specifically, the raw sadness and raw fear themes are displayed with supporting music backgrounds to help trigger the respective emotions well. The stimuli themes used in this study, along with their functions sorted in order, are presented in Table 2. Each stimulus has its specific function related to the attachment system, which causes a varied response in each attachment style.

**Table 2:** Attachment-based theme stimuli.

Stimulus Theme, Objective, Relationship	Stimulus Sample
Theme: Exploration Objective: Depends on Attachment Style Relationship Type: Self-Relationship	
Theme: Attuned Mother-Child Objective: Soothing (Attachment Deactivation) Relationship Type: Relationship with Mother	
Theme: Attuned Father-Child Objective: Soothing (Attachment Deactivation) Relationship Type: Relationship with Father	
Theme: Family Connection Objective: Soothing (Attachment Deactivation) Relationship Type: Relationship with Parents	
Theme: Attuned Couple Objective: Soothing (Attachment Deactivation) Relationship Type: Relationship with Romantic Partner	
Theme: Attuned Friendship Objective: Soothing (Attachment Deactivation) Relationship Type: Relationship with Close Friend	
Theme: Raw Fear Objective: Depends on Attachment Style Relationship Type: Self-Relationship	
Stimulus Theme, Objective, Relationship	Stimulus Sample
Theme: Child Solitude Objective: Depends on Attachment Style Relationship Type: Self-Relationship	
Theme: Vulnerable Baby Objective: Depends on Attachment Style Relationship Type: Self-Relationship	
Theme: Unresponsive Mother Objective: Stressing (Attachment Activation) Relationship Type: Relationship with Mother	
Theme: Violent Father Objective: Stressing (Attachment Activation) Relationship Type: Relationship with Father	
Theme: Couple Separation Objective: Stressing (Attachment Activation) Relationship Type: Relationship with Romantic Partner	
Theme: Raw Sadness Objective: Depends on Attachment Style Relationship Type: Self-Relationship	
Theme: Death or Loss Objective: Depends on Attachment Style Relationship Type: Self-Relationship	

Each person has his/her own default expression, called the baseline expression. For example, someone may look happy all the time because his/her lip corners tend to pull up, showing a smiling face. On the other hand, someone may also look sad all the time because of the loose eyelids shape. To help the annotator differentiate the baseline expressions from expressions that show emotion, we need to collect the subject's baseline traits before stimuli exposure is given. Based on the advice from expression and behavior analyst, we obtained baseline traits information by asking subjects to introduce themselves and tell us about their embarrassing personal past experiences prior to video and audio data collection. Telling past experience is considered to be effective in bringing out all the distinctive baseline expressions of each subject.

We collected pictures that we considered relevant for each stimulus theme from iStockPhoto^1^ by using the additional keywords “Asian” or “Indonesian” in each stimulus theme, for example “Asian attuned mother-child”. All stimulus images were obtained from iStockPhoto, except for the raw fear and raw sadness themes, which were obtained from Google image search results. This is in contrast to the stimuli selection in Parra et al., [8], which the stimulus pictures were obtained from three large picture databases on emotion: the Nencki Affective Picture System (NAPS), the International Affective Picture System (IAPS), and the Geneva Affective Picture Database (GAPED). We did not search pictures from those databases because most of their pictures do not represent Indonesian or Asian faces and cultures.

Since iStockPhoto did not categorize the pictures as we expected, we need to determine which pictures best represent the theme we have defined. Therefore, we conducted a small survey to select pictures that best fit the theme and can effectively stimulate the intended responses from the target data collection subjects. Three respondents with the same criteria as the data collection subjects were randomly selected to participate in the survey by sorting the pictures that best described each theme. We extracted the same best-choice pictures from each subject for the data collection stimuli set.

### 4.2 Subject recruitment

The subject recruitment process was carried out by distributing posters online. Using the Snowball sampling technique, this poster was shared on various social media platforms, including Twitter, Instagram, and Whatsapp groups. It contained information about the volunteer needs of research subjects. In detail, it contained brief information about the participant criteria, what forms of participation were needed in the observation, the criteria for place and equipment that needed to be prepared by subjects, information that the data to be taken would only be used for non-commercial research purposes, and a link referred to the page for filling out the form of willingness to participate in this study. Subjects provided their contact information and chose the most convenient time to participate in the experiment.

The target subjects are young adults with an age range of 18-29 years old. In the subject recruitment process, we did not determine specific criteria regarding mental health status because our primary focus is on the attachment styles classification of young adults in general. We also expected the subjects to vary as much as possible regarding age, ethnicity, relationship, recent education, occupation, and parental status. The subject's willingness to participate in this study was indicated by filling in their required personal data and signing the informed consent form.

Referring to the study by Parra et al., [7] in which 59 subjects participated, we initially intended to collect 60 subjects. However, we assumed this number was still inadequate for training the machine learning model, as it required a large amount of data. Based on these considerations, we managed to collect data from 95 subjects. However, we did not include the data of 8 subjects due to irrelevant responses to the stimuli given, and poor video and audio quality. In the end, the data of 87 subjects was sufficient to be used in training the machine learning model. In addition, we also collected 3 subjects with the same criteria to be included in the stimuli selection survey.

### 4.3 Self-report questionnaire completion

Each subject needed to provide their ratings on the ECR-RS questionnaire [1] prior to the experimental data collection process. Upon the use on Indonesia young adults, this questionnaire needs to be translated into Bahasa Indonesia. We have gotten permission from Fraley to translate the ECR-RS original version to Bahasa Indonesia. The translation process was conducted by four linguists to ensure validity and keep the original meaning of each statement well conveyed. Then, the translation results from all linguists were consulted with experts for the readability test. Table 3 displays all items of the original ECR-RS and Bahasa Indonesia version.

**Table 3:** Original and Bahasa Indonesia version of ECR-RS questionnaire.

#	Statement (Original Version)	Statement (Bahasa Indonesia Version)
1	It helps to turn to this person in times of need (R)	Pada saat saya membutuhkan, berpaling pada orang ini bisa membantu (R)
2	I usually discuss my problems and concerns with this person (R)	Saya biasanya menceritakan masalah dan kekhawtiran saya pada orang ini (R)
3	I talk things over with this person (R)	Saya membicarakan banyak hal dengan orang ini (R)
4	I find it easy to depend on this person (R)	Saya merasa mudah untuk bergantung pada orang ini
5	I don't feel comfortable opening up to this person	Saya tidak merasa nyaman membuka diri pada orang ini
6	I prefer not to show this person how I feel deep down	Saya memilih untuk tidak menunjukkan apa yang saya rasakan di lubuk hati terdalam pada orang ini
7	I often worry that this person doesn't really care for me	Saya sering khawatir orang ini tidak sungguh-sungguh peduli dengan saya
8	I'm afraid that this person may abandon me	Saya takut bahwa orang ini akan mengabaikan/meninggalkan saya
9	I worry that this person won't care about me as much as I care about him or her	Saya khawatir orang ini tidak peduli pada saya sepeduli saya padanya

ECR-RS contains nine items applied for four specific relationships (i.e., mother, father, romantic partner, and best friend), so the total items are 36. The general attachment representation is obtained by averaging all specific relationship scores. For each specific relationship, there are two scores, one for attachment-related anxious and the other for attachment-related avoidance. The anxious score is computed by averaging items 7-9. The avoidance score is computed by averaging items 1-6, with reverse keying items 1, 2, 3, and 4. If the anxious score is greater than avoidance score, the respective subject can be categorized as anxious, and vice versa. We can categorize the subject as secure if both scores are relatively similar. Based on the guidance of psychology experts, we use a Likert scale of 1-6 (strongly disagree – strongly agree). The application of even numbers scale is because some subjects tend to choose a neutral scale when in doubt, which can complicate the inference process of the score results. The Indonesia version of the ECR-RS questionnaire has undergone a validity and reliability testing process for each specific and general relationship in 50 sample subjects. The results show that all items are valid and reliable for assessing attachment style in young adults.

This questionnaire was administered to each subject online along with the informed consent. It is important for each subject to complete each item based on his/her current condition, rather than what (s)he believes is ideal. There are specific instructions for relationships with parents and romantic partner. In the relationship with mother and father, each subject needs to rate based on their feelings toward their relationship with mother and father figures or figures they consider mother and father. In a relationship with a romantic partner, there are several conditions, 1) if the subject is currently in a romantic relationship (dating or married), they need to give a rating that best describes their feelings toward their relationship with their partner, 2) if the subject is not currently in a romantic relationship, then they need to provide a rating regarding how they felt in their last romantic relationship, 3) if the subject has never been in a romantic relationship at all, then they can respond by referring to their preferences for future romantic relationships.

### 4.4 Video and audio data collection

Since the data collection was done during the COVID-19 pandemic, we collected the data online using a video conference application, Zoom. Each participant had a 1:1 session via Zoom with one of our research team whom clinical psychology experts have trained. The training includes how to conduct an effective projective-based experiment and what to anticipate and do when there are unexpected emotional reactions. We recorded all processes during the experiment, focusing on facial expressions and speech signals during exposure to attachment-themed stimuli. To support the data collection process run smoothly, each subject prepared a laptop equipped with a webcam and audio that worked well, a Zoom application installed, mics/headphones/headsets, and a smooth internet connection. The experiment could take place anywhere as long as participants ensured they were in a quiet room with bright light exposure and not accompanied by anyone.

The experiment was divided into two stages, exposure, and response sequentially for each stimulus with a total duration of ± 30-45 minutes. The presentation of stimuli or instruction related to them was available in a video presentation with an automatic timer during the process. Besides, our team member did not activate the video, any help and urgent instruction were informed by voice only. These rules were made to create a calm atmosphere so that the participant was not affected by our team member's expressions and behavioral cues, could be more relaxed, and did not feel embarrassed or nervous because of being watched. In addition, during the experiment, participants also needed to maintain the right distance between themselves and the laptop so that the camera could capture all facial components, and nothing was blocking the face.

At the exposure stage, each subject was instructed to only focus on observing the image being presented. There were 14 image themes in total, each of which was displayed respectively in ± 30 seconds except stimulus number 7 (raw fear) and 13 (raw sadness), which were presented with background music in ± a minute 30 seconds. Every single image that had been presented would be followed by the response stage indicated by an automatic transition to the instruction: “tell us how you feel, or experience related to the picture in a minute”. In this session, the subjects were given 1 minute to answer the question by telling their feelings or experiences related to the activity or relationship in the picture they observed before. This process repeated until the last stimulus image.

### 4.5 Dataset labeling and annotation

We annotated emotion and attachment labeling based on attachment annotation criteria that we compiled from general and special characteristics according to attachment theory, and emotion representation of behavior during assessment. The formulation of these criteria has been reviewed and approved by a professional psychologist. The attachment annotation criteria are shown in Table 4. A subject may be classified into a particular attachment class even when he or she only exhibits an emotion among some emotions mentioned.

**Table 4:** Attachment Annotation Criteria

Stimuli	Behavioral Characteristics		
	Secure	Anxious	Avoidance
Exploration	• Have a great willingness to explore [9], [10]. • Have higher achievement motivation (less fear of failure) [11]. • are more open to exploration compared to insecurely attached one [9]. • Dominant emotion: happy, less fear.	• Show less desire to explore [9]. • Over-dependence and lack of self-confidence. • are more curious and have positive mind on exploration than avoidance [10]. • Tend to have negative assumption on environmental exploration [12]. • Dominant emotion: Happy, fear (environment).	• Have less desire to explore and less curiosity [12]. • Less likely to engage in exploration. • Tend to inhibit attachment needs by using exploration to avoid social interaction [12]. • Dominant emotion: neutral, fear (social).
Child Solitude	• Feel comfortable with solitude. • When alone, they do not feel lonely because they have positive social support perceived. • Can make good use of their alone time. • Dominant emotion: happy, neutral.	• is associated with loneliness. • Tend to worry excessively on attachment figure unavailability [13]. • Perceive unavailability of others as rejection and abandonment. • Exaggerate their dissatisfaction and neediness [4]. • Dominant emotion: sad, anger.	• is associated with loneliness but not admitting a need for care. • Feel less directly or less consciously irritated by the unavailability of others. • May feel bored and meaningless during solitude time. • Dominant emotion: neutral.
Raw Fear	• is slow to recognize threats and make effective decisions. • Tend to look for and turn to others first rather than finding a solution.	• Hypervigilance. • React more sensitively and quickly upon unfamiliar and ambiguous danger signals [14]. • Hypervigilance	• Tend to develop fight-or-flight behavior [14]. • Focus on discovering the effective solution. • Dismiss threats and avoid interaction.
Raw Fear	• At first, they may show surprise and fear, but will slowly feel better due to good emotion regulation and self-reassurance. • Dominant emotion: surprise, fear.	• Actively seek the help of others by exaggerating their emotions. • Dominant emotion: surprised, fear.	• Compulsively self-reliance (show not being affected, not wanting to appear vulnerable and insufficient) [4]. • Dominant emotion: neutral.
Raw Sadness	• Tend to turn and stay together with their loved ones. • May feel sad and disappointed but not to the point of emotional change to anger. • Able to self-soothe, regulate emotion, and reappraise in a positive way [4]. • Dominant emotion: sad, happy.	• Seek attention, care, and support from significant others by overacting their emotions. • Upon unattainable attachment figures, they tend to overanalyze and assume it as a sign of rejection and ignorance. • People with anxious histories may feel sad, hurt, and angry when receive the same treatment again. • Dominant emotion: sad, anger.	• Tend to suppress emotion as a self-protective response [4]. • Tend to not show their vulnerability and the need of intimacy with significant others. • Tend to show that they are sufficient and do not need others. • Dominant emotion: neutral.
Death or Loss	• May feel sad and disappointed but not to lead to anger. • Cope better with loss. • Able to self-soothe and reappraise positively. • Dominant emotion: sad, happy.	• is difficult to accept and make peace with death or loss [4]. • Tend to blame themselves for abandonment of others. • Dominant emotion: sad, anger.	• Tend to not show their vulnerability explicitly. • Tend to suppress their emotion. • Have a great fear of death [4]. • Dominant emotion: neutral.
Vulnerable Baby	• May feel sad and concerned at first but have a hopeful positive appraisal. • Dominant emotion: sad, happy.	• Tend to feel sad and worry excessively. • Dominant emotion: neutral, sad.	• Tend to show little or no concern because it may reactivate their attachment. • Dominant emotion: neutral.
Attuned Mother-Child, Attuned Father-Child, Family Connection, Attuned Couple, Attuned Friendship. (*Five kinds of attachment relationship differing only on attachment figures involved*).	• are more cheerful than either anxious or avoidant [15]. • See the respective attachment figure as reachable, available, and responsive. • View themselves as good enough and worthy of love. • Dominant emotion: happy.	• High levels of sadness and worry that they will be abandoned and neglected later. • Assume others are not always available for them all the time. • Act clingy to gain attention from attachment figure. • Dominant emotion: happy, worry/fear, sad, anger.	• Uncomfortable with closeness and intimacy. • Assume that others are not reliable or available when needed. • Tend to think highly of themselves and look down on others. • Dominant emotion: neutral, contempt [15].
Unresponsive Mother	• Tend to have a healthy relationship with parents. • Able to self-soothe, regulate emotion, and reappraise the unresponsive mother positively. • Dominant emotion: neutral, sad, happy.	• Higher level of sadness and anxiety. • Feel guilty when they assume that rejection, unavailability, conflict, and unresponsive acts of others are due to their attitudes. • Associated with anger and disgust [15]. • Act clingy to gain attention from others. • Dominant emotion: sad, anger, disgust.	• Tend to withdraw, repress their need, and cope on their own. • Seek emotional support through indirect way, e.g., hinting, complaining, sulking. • Tend to think highly of themselves (feel that they can do anything well on their own). • Dominant emotion: neutral, anger, disgust, contempt [15].
Violent Father	• Tend to have a healthy relationship with parents. • If the father figure ever shows abusive act (e.g., giving advice in harsh words), they may reappraise it positively as a form of care. • Dominant emotion: neutral, sad, happy.	• is associated with childhood physical abuse and neglection [16]. • Afraid of any violent behavior and feared that it is all their own fault that caused them to be abandoned. • Feel irritated when received abusive act even they do not cause any trouble. • Dominant emotion: fear, sad, anger.	• is associated with physical abusive act [16]. • Feel inadequate and worthless. • See themselves as self-sufficient, deny vulnerability, and avoid close relationships. • Dominant emotion: neutral, anger, disgust, contempt [15].
Couple Separation	• May feel sad and disappointed but not to the point of emotional change to anger. • Cope better with separation because they allow themselves to feel sad. • Able to regulate emotion and reappraise in a positive way. • Dominant emotion: sad, happy.	• Show angry protests, lost sense of identity, and preoccupation with the lost partner [17]. • Try to mend the broken relationship and beg not to be abandoned by acting irritatingly [4]. • React with strong self-blame and intense distress. • Dominant emotion: sad, anger, disgust.	• Less inclined to seek support and more prone to cope with the breakup alone [17]. • Tend to avoid new romantic commitments. • Suppress separation-related thoughts [4]. • Dominant emotion: neutral.

In developing attachment annotation criteria, we also refer to individual differences discourse in the AAI procedure based on Grice's conversational maxim [18] for identifying paralinguistic characteristics of our data presented in Table 5. Based on the information presented in the table, a verbatim report of AAI can be classified as secure when it follows all points of Grice's maxim. While insecure anxious text violates the maxim of quantity, relation, and manner. It violates quantity because mostly anxious people answer in excessively long response by adding unnecessary details not asked in the topic, which also violates the maxim of relation and manner. For example, when anxious person is asked about the query of relationship with their mother in childhood, they address the response to their current relationship with their mother or their relationship with their children. In contrast, insecure avoidance text violates the maxim of quality and quantity. Regarding quality, most avoidance persons describe parents with highly positive generalized representations contradicting the specific episodes recounted. They show the inconsistent response as an attempt to inhibit the memories of events that evoke attachment activation from affecting them. Avoidance interview text also violates quantity in terms of excessively short responses, either because of repeated insistence on lack of childhood memories or refusal to discuss by saying “I do not remember”.

**Table 5:** Individual differences in AAI based on Grice's maxim.

Attachment Style	Grice's Maxim			
	Quality	Quantity	Relation	Manner
Secure	Response is truthful, clear, and reasonably consistent.	Response is reasonably succinct.	Response is relevant to the topic asked.	Response is clear and orderly.
Anxious	Response is truthful, clear, and reasonably consistent.	Response is excessively long (contains unnecessary details).	Response is irrelevant to the topic asked.	Response is grammatically entangled, may unfinished, and contains vague terms.
Avoidance	Response is internally inconsistent (generalized representations of history are contradicted with episodes recounted).	Response is excessively short (either because of insistence on absence of memory or refusal to discuss).	Response is relevant to the topic asked.	Response is clear and orderly.

As mentioned before, the emotion labels are assigned in a multi-label manner for all stimuli on each exposure video, response video, and speech audio. Using Krippendorff's alpha (α), we measured the inter-rater agreement on the emotion labeling on 43 subjects’ data. It is a measurement that compares the observed disagreement with the expected disagreement. We choose this approach since it is reliable for handling multi-label data, e.g., [19,20]. We obtained Krippendorff's alpha agreement of 0.9099, 0.6489, and 0.8115 on exposure-only, response-only, and exposure-response, respectively. Agreement in exposure data is greater than in response data because most subjects tend to focus on paying attention to the stimuli, which results in the appearance of neutral as the dominant expression. A genuine expression that appears as an early response to a stimulus generally only lasts for a short time and is not complex, resulting in a high agreement between the two raters in exposure data. This differs from when subjects recount their experience, which simultaneously encourages the elicitation of several emotions. We assume that two factors may influence inter-rater disagreement. First, different annotators' preferences in giving ratings: e.g., the first annotator who pays attention to the emotions that appear at a specific time range differs from the second annotator who tends to label emotion in general terms of time. Second, different annotators' personal experiences and values. Differences in life experience may bring subjectivity in the way someone defines categorization. Agreement on the combination of exposure and response annotations is quite good. For attachment labels which are assigned in a single label for each subject, we obtained a Cohen's Kappa agreement score of 0.7577.

## Ethics Statements

Informed consent was acquired from each subject before participating in the behavioral assessment. We informed subjects that their participation is voluntary, they are free to refuse to participate, and if they decide to participate, they are free to pause or withdraw at any time without penalty. All collected data from the voluntary subjects are presented anonymously. We ensured that the subject's personal information (e.g., name, address, mobile number) is kept confidential and can only be accessed by our research team. The whole dataset collection procedure was ethically approved by the ethical committee of the Faculty of Psychology, Universitas Indonesia (approval letter code: 120/FPsi.Komite Etik/PDP.04.00/2021).

## CRediT authorship contribution statement

**Tusty Nadia Maghfira:** Conceptualization, Methodology, Formal analysis, Investigation, Resources, Data curation, Writing – original draft. **Adila Alfa Krisnadhi:** Conceptualization, Methodology, Resources, Supervision, Writing – review & editing. **T. Basaruddin:** Conceptualization, Supervision, Resources, Funding acquisition, Writing – review & editing. **Sri Redatin Retno Pudjiati:** Conceptualization, Methodology, Resources, Supervision.

## Data Availability

The Indonesian Young-Adult Attachment (IYAA) Dataset: A Multimodal Behavioral Young-Adult Attachment Assessment (Original data) (Zenodo).

## Appendix Supplementary materials

Image, application 1

## Footnotes

1 https://www.istockphoto.com/.
