# Supplementary material for: The Indonesian Young-Adult Attachment (IYAA): An audio-video dataset for behavioral young-adult attachment assessment
Source: Data Brief. 2023 Sep 21;50:109599. doi: 10.1016/j.dib.2023.109599 (PMC10539883; doi:10.1016/j.dib.2023.109599)
Supplement: Supplementary file 1 [file mmc1.pdf]

DATA USE AGREEMENT FOR IYAA DATASET

This Data Use Agreement ("Agreement"), effective as of ____________ (Effective Date), is entered into by and between ___________________ (Recipient) and ___________________ (Covered Entity). The purpose of this Agreement is to provide Recipient with access to the Indonesian Young-Adult Attachment (IYAA) Dataset for use in the following titled Research Project: _________________________________ (Research Title) under the direct supervision of ___________________ (Principal Investigator) in accord with the Indonesian Data Protection Law and other relevant laws.

1. Definitions.
   Covered Entity: IYAA Dataset Development Team – who conducted the original study from which the data were generated. The team consists of the following researchers:
   i. Tusty Nadia Maghfira – Faculty of Computer Science, Universitas Indonesia
   ii. Adila Alfa Krisnadhi – Faculty of Computer Science, Universitas Indonesia
   iii. T. Basaruddin – Faculty of Computer Science, Universitas Indonesia
   iv. Sri Redatin Retno Pudjiati – Faculty of Psychology, Universitas Indonesia
   Recipient: the person who submits a request for access to the IYAA Dataset, and (s)he is considered the research manager of the Research Project.
   Principal Investigator: the person who leads and possesses the highest supervisory role in the Research Project.
   Disclosure: the release, transfer, provision of access to, or divulging in any manner of information outside the entity holding the information.
   Individuals: all persons whose private and non-private data are collected in the IYAA Dataset and subject to protection of information according to applicable law.
   Subcontractor: a person to whom a recipient delegates a function, activity, or service, other than in the capacity of a member of the workforce of such recipient.
2. Preparation of the IYAA Dataset. Covered Entity shall prepare and furnish the IYAA Dataset to Recipient in accord with the Indonesian Data Protection Law and other relevant laws.
3. Data Fields in the IYAA Dataset.
   a. The furnished IYAA Dataset shall include, for each Individual:
      - a set of annotated facial expression videos obtained when the Individual is exposed to stimuli;
      - a set of annotated facial expression and speech videos obtained after the Individual is exposed to stimuli;
      - a set of Experiences in Close Relationship – Relationship Structures (ECR-RS) questionnaire responses;
      - an attachment style label.
   b. The Covered Entity shall not provide any amendment and reduction of data as mentioned in this Agreement after access to the data is granted to the Recipient.

4. Responsibilities of Recipient. Recipient agrees to:
   a. Use or disclose the IYAA Dataset only as permitted by this Agreement or as required by law;
   b. Use appropriate safeguards to prevent use or disclosure of the IYAA Dataset other than as permitted by this Agreement or required by law;
   c. Report to Covered Entity any use or disclosure of the IYAA Dataset of which it becomes aware that is not permitted by this Agreement or required by law, including the presence of prohibited identifiers in the IYAA Dataset;
   d. Require any team member of the Research Project mentioned in this Agreement, who receive or have access to the IYAA Dataset to agree to the same restrictions and conditions on the use and/or disclosure of the IYAA Dataset that apply to Recipient under this Agreement;
   e. Require any Subcontractors or agents associated with the Recipient, who also need access to the IYAA Dataset, to obtain agreement directly from the Covered Entity, and;
   f. Not use the information in the IYAA Dataset, alone or in combination to identify or contact the Individuals as defined in section 1.
5. Permitted Uses and Disclosures of the IYAA Dataset. Recipient may use and/or disclose the IYAA Dataset restricted only to the Research Project that is stated at the beginning of this Agreement and described in the Appendix of this Agreement.
6. Term and Termination.
   a. Term Commencement. The term of this Agreement shall commence as of the Effective Date.
   b. Term Termination. This Agreement shall terminate one year after the Effective Date. Should the Recipient desire to keep the IYAA Dataset for a longer period, a justification in writing should be made to the Covered Entity. Upon the termination of this Agreement, the Recipient shall destroy any copy of the IYAA Dataset in possession.
   c. Termination by Recipient. Recipient may terminate this Agreement at any time prior to the term termination date as defined in section 6(b), by notifying the Covered Entity in writing. Upon termination of this Agreement, the Recipient shall destroy any copy of the IYAA Dataset in possession.
   d. Termination by Covered Entity. The Covered Entity may terminate this Agreement at any time prior to the term termination date as defined in section 6(b), by providing a 30-day written notice to the Recipient.
   e. For Breach. The Covered Entity shall provide a written notice to Recipient within ten (10) days of any determination that Recipient has breached a material term of this Agreement. The Covered Entity shall afford the Recipient an opportunity to cure said alleged material breach upon mutual agreeable terms. Failure to agree on mutually agreeable terms for cure within thirty (30) days shall be grounds for the immediate termination of this Agreement by the Covered Entity.
   f. Effect of Termination. Sections 1, 4, 5, 6(f) and 7 of this Agreement shall survive any termination of this Agreement under subsections d, or e.

7. Miscellaneous.
   a. Change in Law. The parties agree to negotiate in good faith to amend this Agreement to comport with changes in Indonesian Data Protection Law and other relevant laws that materially alter either or both parties' obligations under this Agreement. Provided however, that if the parties are unable to agree to mutually acceptable amendment(s) by the compliance date of the change in applicable law or regulations, either Party may terminate this Agreement as provided in section 6.
   b. Construction of Terms. The terms of this Agreement shall be construed to give effect to applicable interpretative guidance regarding the Indonesian Data Protection Law and other relevant laws.
   c. No Third-Party Beneficiaries. Nothing in this Agreement shall confer upon any person other than the parties and their respective successors or assigns, any rights, remedies, obligations, or liabilities whatsoever.
   d. Counterparts. This Agreement may be executed in one or more counterparts, each of which shall be deemed an original, but all of which together shall constitute one and the same instrument.

IN WITNESS WHEREOF, each of the undersigned has caused this Agreement to be duly executed in its name and on its behalf.

COVERED ENTITY

Name: _________________________
Signature: ______________________
Date: _____________

RECIPIENT

Name: _________________________
Signature: ______________________
Date: _____________

Acknowledgement. This Data Use Agreement (DUA) is adapted from the Data Use Agreement for Limited Data Sets (DUA-LDS) format by Harvard Catalyst Data Protection subcommittee, which is a subcommittee of the Regulatory Knowledge & Support Program and affiliated with Harvard Catalyst | The Harvard Clinical and Translational Science Center. The original DUA-LDS is accessible from https://catalyst.harvard.edu/publications-documents/data-use-agreement/. The content of this DUA is solely the responsibility of the authors and does not necessarily represent the official views of Harvard Catalyst, Harvard University and its affiliated academic health care centers, or the National Institutes of Health.

Appendix - Terms of Access

Instructions. If the Research Project is a thesis, please fill in section 1 and 3 only. Otherwise, fill in section 2 and 3 only. Do **not** fill in section 1 and 2 simultaneously.

1. **Thesis Project**

   Title:____

   Recipient (as defined in section 1 of the main body of the Agreement):

   Name:_________________
   Email:______________________
   Affiliation: ______________

   Student (only if the student is different from the Recipient):

   Name:_________________
   Email:______________________
   Affiliation: ______________

   Primary Thesis Supervisor:

   Name:_________________
   Email:______________________
   Affiliation: ______________

   Additional Team Members Involved in the Project: (including the name, email, affiliation)

   1. ___________________________________
   2. ___________________________________
   3. ___________________________________
   4. …

2. **Non-Thesis Research Project**

   Title:____

   Recipient (as defined in section 1 of the main body of the Agreement):

   Name:_________________
   Email:______________________
   Affiliation: ______________

   Principal Investigator:

   Name:_________________
   Email:______________________
   Affiliation: ______________

   Team Members Involved in the Project: (including the name, email, affiliation)

   1. ___________________________________

2. ____
3. ____
4. …

3. **General Project Information**

Description of the research project and how one intends to use the data:

(max 500 words) (a number of original references can be provided)

____
____
____
____
____

Specify the required variables from IYAA Dataset to carry out the Research Project

1. __________________
2. __________________
3. __________________
4. …

Research Project URL Link (if it is available):

____
